# Epidemiological, clinical and biological profile of neuromeningeal cryptococcosis among people living with HIV in Kinshasa, Democratic Republic of Congo

**DOI:** 10.11604/pamj.2020.37.302.20521

**Published:** 2020-12-02

**Authors:** Bive Zono, Erick Kamangu, Hippolyte Situakibanza, Evelyne Amaela, Ben Bepouka, Marcel Mbula, Jean-Marie Kayembe, Georges Mvumbi, Marie-Pierre Hayette

**Affiliations:** 1 Service of Molecular Biology, Department of Basic Sciences, Faculty of Medicine, University of Kinshasa, Kinshasa, Democratic Republic of Congo,; 2 Service of Infectious Diseases, Department of Internal Medicine, Faculty of Medicine, University of Kinshasa, Kinshasa, Democratic Republic of Congo,; 3 Service of Pneumology, Department of Internal Medicine, Faculty of Medicine, University of Kinshasa, Kinshasa, Democratic Republic of Congo,; 4 Service of Molecular Biology, Department of Clinical Microbiology, University of Liege, Liege, Belgium

**Keywords:** Epidemiology, clinical signs, neuromeningeal cryptococcosis, HIV, Democratic Republic of Congo

## Abstract

Neuromeningeal cryptococcosis (NMC) is one of the most frequent opportunistic infections (OI) in Human Immunodeficiency Virus (HIV) infection. In Kinshasa, the latest data on cryptococcosis were published in 1996. The objective was to describe the epidemiological, clinical and biological profiles of NMC in HIV-infected people living in Kinshasa. This is a descriptive study based on the medical records of patients who attended three clinics in Kinshasa between January 1 ^s^^t^ 2011 and December 31^st^ 2014. Only the medical records of HIV-infected people presenting the NMC were reviewed. During the 4 year-period of the study, 261 HIV-positive patients presented to the clinics for neuromeningeal syndrome, including 23 with NMC. The global prevalence of NMC was 8.8% for the three clinics. The mean age was 42.8 ± 9.5 years, with male predominance (65.2%). The main symptoms were headache (73.9%), neck stiffness (60.9%), fever (47.8%), and coma (47.8%). Biological records were as follows: median CD4 cell count was 79 cells/mm^3^; cerebrospinal fluid (CSF) was clear for 56.5% of the cases with predominance of neutrophils in 73.9%. The outcome was fatal in 34.8% of cases. The prevalence and therapeutic outcome of NMC show that it constitutes a non-negligible OI in Kinshasa, especially in HIV-infected people at the AIDS stage. As HIV-infected people with severe immunosuppression are the most affected by NMC, active preventive measures should benefit this vulnerable category of people.

## Introduction

Neuromeningeal cryptococcosis (NMC) is one of the most frequent opportunistic infections (OI) in term of morbidity and mortality in advanced stages of HIV infection [1]. The causal agent of NMC is a fungus belonging to the species complex *Cryptococcus neoformans/C. gattii*. Rarely met until the 1980s, this infection has since seen its incidence increase considerably with the emergence of the AIDS pandemic [2]. Despite the availability of antiretroviral (ARV) drugs against HIV infection, the mortality rate of NMC within 10 weeks after the start of antifungal therapy varies from 20% to 50% in sub-Saharan Africa [3,4]. A survey conducted in 17 European countries by the European Confederation of Medical Mycology (ECMM) found that cryptococcosis was associated with HIV infection in 77% (435/565) of cases [5]. Also, among People Living With HIV (PLWHIV) in Ivory Coast, Kassi Kondo [6] estimated prevalence of NMC at 3.6%; in Togo, this prevalence was estimated at 2.9% [7]. In the Democratic Republic of Congo (DRC), the Demographic and Health Survey 2013-2014 (EDS DRC II) estimated that 1.2% of the general population aged 15-49 are infected with HIV and, according to projections based on Spectrum 5.41, the number of PLWHIV in the DRC was estimated to be 381,187 by the end of 2016, of which only 121,762 would be on ARV Treatment (ART) and 20,854 would die [8]. Among these PLWHIV, more than half do not have access to ART and are thus exposed to all the complications related to the natural history of the disease, including OI [9]. In addition to HIV infection, other immunosuppressive situations have been associated with an increased risk of contracting cryptococcosis [10]. In the DRC, the hospital frequency of cryptococcosis ranged from 19% in 1994 to 11% in 2013 [11,12]. In Kinshasa, the latest available data on cryptococcosis dates back to 1996, yet the extent of HIV infection in this population is constantly increasing [13]. The lack of an up-to-date epidemiological and clinical data on this fungal disease among HIV patients in the DRC in general and specifically in Kinshasa motivated the initiation of the present work. The objective of this study was to describe the epidemiological, clinical characteristics and the profile of biological analyses during NMC among PLWHIV who attended clinics in Kinshasa.

## Methods

**Study frame:** this is a descriptive study based on the medical records of HIV-positive patients who attended three hospitals located in different districts of Kinshasa, between January 1^st^ 2011 and December 31^st^ 2014. Among these hospitals, the University Clinic of Kinshasa (CUK) was the only tertiary level hospital with an active line of 124 HIV-positive patients. The 2 other hospitals were the Kinshasa Reference General Provincial Hospital (HPGRK) and the Monkole Hospital Center (CH Monkole) with a significant active patient population of 1717 and 1138 PLWHIV, respectively.

**Inclusion criteria:** the present study took into account the HIV-positive patients in an exhaustive way, whether they were followed regularly or not. Were included in the study, patients who consulted with a clinical presentation of meningoencephalitis, particularly those with NMC confirmed by the direct test using India ink and having been hospitalised in the selected hospitals during the period of study.

**Data collection:** the data were collected using a survey form. The following variables were considered in this study: socio-demographic (sex, age and marital status), clinical (motive for consultation, medical history, physical examination data, duration and outcome of hospitalisation) and biological (CD4 cell count as well as direct India ink staining, biochemistry, cytology and Sabouraud culture of cerebro-spinal fluid) variables.

**Statistical analyses:** the data were entered on an excel spreadsheet and then exported to SPSS 26.0 (statistical package for the social sciences 26.0) for descriptive analysis. Quantitative variables were presented as proportions (%), means and standard deviations. Qualitative variables were given in the form of proportions (%).

**Ethical considerations:** this work has been performed in strict compliance with ethical rules by guaranteeing the anonymity and the safety of patients. The data collected were kept and manipulated by the research team alone.

## Results

### Epidemiological characteristics

**Frequency of neuromeningeal cryptococcosis:** out of 261 PLWHIV included in the study, 23 presented with NMC, an overall prevalence of 8.8%. The relative prevalence was higher at HPGRK with 15 cases out of 98 patients included (15.3%) followed by CUK (6 out of 65 patients included, 9.2%) and CH Monkole (2 out of 98 patients included, 2%).

**Sociodemographic data:** in this study, men were more affected than women (65.2%) with a sex ratio M/F of 1.9. The mean age of patients was 42.83 ± 9.5 years with an age range from 31 to 62 years. The 35 to 44 age group was the most affected, at 34.8%. The highest frequency of the disease was observed among married, 39.1% (Table 1).

**Table 1:** socio-demographic variables of patients

Variable	Female n (%)	Male n (%)	Total n (%)
**Sex**			
	8 (34.8)	15 (65.2)	23 (100)
**Age group (year)**			
25 – 34	2 (8.7)	3 (13.0)	5 (21.7)
35 – 44	3 (13.0)	5 (21.7)	8 (34.8)
45 – 54	1 (4.3)	5 (21.7)	6 (26.1)
55 – 71	2 (8.7)	2 (8.7)	4 (17.4)
**Civil status**			
Married	2 (8.7)	9 (39.1)	11 (39.1)
Single	1 (4.3)	5 (21.7)	6 (26.1)
Divorced	2 (8.7)	1 (4.3)	3 (13.0)
Widower	3 (13.0)	0	3 (13.0)

**Clinical data:** headache (73.9%), fever (47.8%) and coma (39.1%) were the most common motives for consultation. Alcohol uptake (26.1%) and hypertension (13%) were the most commonly reported antecedents in these patients. The mean duration of hospitalisation was 15.3 ± 10.4 days with extremes of 2 and 36 days; the majority of patients had stayed either 1 to 7 days or 15 to 21 days in the hospital (30.4% each). After treatment, survival rate was 60.9%, 3.4% were lost to follow-up, and the outcome was fatal in 34.8% (Table 2). After physical examination, 52.2% of patients were lucid, 60.9% had stiff neck. Facial paralysis, monoparesis of a member, hemibody hypotonia and presence of cutaneous-plantar reflexes were all found in 8.7% of cases (Table 3). Antifungal therapy consisted mainly of fluconazole alone in 82.6% (19/23) of the cases, at a dosage of 400-800 mg/day during the 14 days of the attack phase and then 200 mg/day during the maintenance phase to immune restoration (CD4 cell count > 100 cells/mm^3^) under ART.

**Table 2:** clinical data (1)

Variable	Female n (%)	Male n (%)	Total n (%)
**Motif for consultation**			
Headache	6 (26.1)	11 (47.8)	17 (73.9)
Fever	3 (13.0)	8 (34.8)	11 (47.8)
Coma	3 (13.0)	6 (26.1)	9 (39.1)
Other	5 (21.7)	3 (13.0)	8 (34.8)
Vomiting	0	4 (17.4)	4 (17.4)
Convulsions	1 (4.3)	2 (8.7)	3 (13.0)
**Antecedents**			
Alcohol	1 (4.3)	5 (21.7)	6 (26.1)
High blood pressure	2 (8.7)	1 (4.3)	3 (13.0)
Tobacco	0	2 (8.7)	2 (8.7)
None	5 (21.7)	7 (30.4)	12 (52.2)
**Duration of hospitalisation (days)**			
1 - 7	4 (17.4)	3 (13.0)	7 (30.4)
8 - 14	2 (8.7)	3 (13.0)	5 (21.7)
15 - 21	0	7 (30.4)	7(30.4)
>21	2 (8.7)	2 (8.7)	4 (17.4)
**Outcome**			
Alive	4 (17.4)	10 (43.5)	14 (60.9)
Dead	3 (13.0)	5 (21.7)	8 (34.8)
Sight loss	1 (4.3)	0	1 (4.3)

**Table 3:** clinical data (2)

Physical examination	Female n (%)	Male n (%)	Total n (%)
**State of consciousness**			
Lucid	4 (17 .4)	8 (34.8)	12 (52.2)
Coma	4 (17.4)	7 (30.4)	11 (47.8)
**Meningeal sign**			
Neck stiffness	5 (21.7)	9 (39.1)	14 (60.9)
Kernig	3 (13.0)	6 (26.1)	9 (39.1)
Brudzinski	2 (8.7)	5 (21.7)	7 (30.4)
**Facial paralysis**	1 (4.3)	1 (4.3)	2 (8.7)
**Motor deficiency**			
Monoparesis	0	2 (8.7)	2 (8.7)
Hemiplegia	0	1 (4.3)	1 (4.3)
**Hypotonia of the hemicorps**	0	2 (8.7)	2 (8.7)
**Skin and plantar reflex**	0	2 (8.7)	2 (8.7)

**Biological data:** the median CD4 cell count in the peripheral blood was 79 (66-105) cells/mm^3^ with a range of 33 to 218 cells/mm^3^. The majority of patients had a CD4 cell count between 50 and 199 cells/mm^3^ (81.8%). In most cases, CSF was clear (56.6%) with predominantly neutrophilic cytorachia (73.9%) and proteinorachia was greater than 45 mg/dl in 6 out of 7 patients from whom proteinorachia data were obtained (Table 4).

**Table 4:** biological data of patients

Variable	Female n (%)	Male n (%)	Total n (%)
**CD4 cell count (cells/mm^3^)**			
<50	0	2 (8.7)	2 (8.7)
50 – 199	7 (30.4)	12(52.2)	19 (82.6)
200 – 349	1 (4.3)	1 (4.3)	2 (8.7)
**CSF appearance**			
Clear	6 (26.1)	7 (30.4)	13 (56.6)
Cloudy	1 (4.3)	4 (17.4)	5 (21.7)
Not reported	1 (4.3)	4 (17.4)	5 (21.7)
**Cytorachia (elements/mm^3^)**			
Neutrophil predominance	5 (21.7)	12 (52.2)	17 (73.9)
Lymphocyte predominance	2 (8.7)	1 (4.3)	3 (13.0)
Mixed	2 (8.7)	1 (4.3)	3 (12.5)
**Proteinorachia (mg/dl)**			
>45	2 (8.7)	4 (17.4)	6 (26.1)
15 – 45	1 (4.3)	0	1 (4.3)
<15	0	0	0
Not reported	5 (21.7)	11 (47.8)	16 (69.6)

## Discussion

The objective of this study was to describe the epidemiological, clinical and biological characteristics of NMC in PLWHIV who attended clinics in Kinshasa. The prevalence of NMC was 8.8% among PLWIHV followed during the four-year period of study. While cryptococcosis is in the process of disappearing completely in developed countries, it still constitutes a burden during HIV infection in African countries [14]. The prevalence from the present study is higher than the 4.6% reported by Mwamba *et al*. in 2013 in Lubumbashi in a prospective study looking for cryptococcal antigen in the blood and the CSF of PLWHIV at the AIDS stage [15]. It is also higher than that reported by Kouakou *et al*. in Ivory Coast in 2016 (2.49%) [16] and that observed in Morocco (1.53%) in a retrospective study of PLWHIV covering a period of 7 years [17]. However, it is lower than that reported in a study conducted in 1996 at the CUK and HPGRK, which are 2 of the 3 sites of the present study, by Situakibanza *et al*. [18] who found cryptococcosis to be responsible for 12.5% of fever cases in PLWHIV. This situation could be explained by the prevalence of HIV infection in the population, estimated at 1.6% in Kinshasa; the inaccessibility of ART for more than half of PLWHIV, as described in the EDS-RDC II 2013 -2014 [8]; and the delay in the diagnosis of HIV infection and its related OI. In addition, at that time, the DRC had not yet adopted the test and treat strategy; ARV treatment was conditioned by certain eligibility criteria that were difficult to meet at the time of diagnosis. It was not until 2015 that the DRC adopted the test and treat strategy [19].

The average HIV-positive young adult male, aged 42.8 ± 9.5 years and married, was the most commonly affected by NMC. These results are linked to the vulnerability to HIV for the young population, who are the most sexually active, a major risk factor for NMC [20]. Our results are consistent with those reported in the literature [20]. Our observation revealed a male predominance (65.2%) with a sex ratio M/F of 1.9. This male predominance is thought to be due to genetic susceptibility linked to a weak phagocytic power and the weak resistance of the male macrophages to *C. neoformans* compared with those of women [21]. In addition, oestrogen may have a protective effect against cryptococcosis [22]. Clinically, headache, fever and loss of consciousness were the most common complaints. However, 52.2% of our patients were afebrile, and had a mild meningeal syndrome limited to neck stiffness in 60.9% of cases. Although the NMC clinical signs are not very specific, fever and headache are often present [20]. In the current study and elsewhere, the predominance of headache and fever has been reported during NMC [15], as well as seizures and motor deficits [23]. The involvement of the cranial nerves, in particular, the facial and oculomotor nerves has also been found [24]. These motor deficits are suggestive of cerebral cryptococcoma (focal lesions) that should be investigated by a brain scan.

From a biological point of view, the CSF was predominantly clear (56.7%) with hyperproteinorachia in 26.1% of cases and, paradoxically, a predominance of neutrophes polymorphonuclear (73.9%). This neutrophilic predominance differs from the literature, which reports the predominance of lymphocytes or a mixed population [25]. In some studies, concomitant Gram and India ink staining of CSF revealed co-infection with *Streptococcus pneumonia* [26]. However, in our study no bacteriological data could be obtained due to lack of availability of the tests. Apart from cytorachia and proteinorachia, our biological results are corroborated by those reported by Chadli in Morocco [17]. CSF culture, a reference test in the diagnosis of NMC due to its high sensitivity and specificity close to 100%, was not performed on the CSF samples taken from these patients, nor was antigen testing undertaken due to a lack of technical means [1,25]. NMC occurred at a very advanced immunodepression stage with a CD4 cell count <200 cells/mm^3^ in 81.9% and for the rest of the cases the CD4 cell count was measured between 200 and 349/mm^3^. These results are in line with data from the literature reporting that PLWHIV south of the Sahara are mostly detected at a time when the CD4 cell count is <200 cells/mm^3^ [23]. Some authors have also noted the unusual occurrence of NMC at the third stage of immunodepression (CD4 cell count between 200 and 349 elements/mm^3^) of HIV disease. Since *Cryptococcus gattii* is present in DRC and is thought to preferentially affect subjects with a good immune status, these cases observed in moderately immunodepressed patients would partially support the presence of this species in the African environment [10]. The viral load, one of the best parameters of the follow-up for the PLWHIV, was not realised in the present study patients for the same reasons evoked above.

Most of the patients received fluconazole alone, 400 to 800 mg/day during the 14 days of the attack phase and then 200 mg/day during the maintenance phase until immune restoration (CD4 cell count > 100 cell/mm^3^) was reached under ART as the main treatment. Other antifungal treatments such as amphotericin B and 5-flucytosine [1] were not widely available in the three clinics. NMC is associated with high and early mortality. Here, 34.8% of our patients died within a mean hospital stay of 15.3 ± 10.4 days. The majority of deaths were observed between 1-7 days. This high mortality can be correlated to the limits of the technical platform for the management of the NMC, in terms of diagnosis, treatment and consultation time. These results are corroborated by other authors [27]. It is important to note that the medium-term prognosis for NMC in HIV depends on the restoration of cell-mediated immunity through ART, the availability of effective antifungals and the permanence of a well-trained caregiver.

## Conclusion

Among the OI occurring during HIV infection, NMC remains a concern due to its prevalence and outcome in Kinshasa. It mainly affects the young adult male with advanced immunodepression. As the clinical signs are not very specific and conventional biology sometimes does not contribute much, the simultaneous realisation of cytorachia and bacteriology of the CSF should be of great value in diagnostic orientation. In addition, the systematisation of an active search for cryptococcal antigens in any HIV-positive patient with very advanced immunodepression, without ignoring the importance of adherence to ART in the primary prevention of cryptococcosis, are ways to be encouraged when caring for these vulnerable people.

### What is known about this topic

- In recent years, the epidemiology of NMC among PLWHIV has been greatly allayed by the availability of triple antiretroviral therapy in some countries;
- The NMC develops most often in the field of dysimmunity, mostly related to HIV and this, at the AIDS stage;
- Cytorachia during NMC is often lymphocytic predominance or mixed, with high proteinorachia and hypoglycorachia.

### What this study adds

- In the major clinics in Kinshasa (DRC), the prevalence of neuromeningeal cryptococcosis among people living with HIV is 8.8%. For a treatment based mainly on fluconazole alone, the therapeutic outcome is fatal in 34% of cases;
- The neuromeningeal cryptococcosis clinic is more marked by headaches (73.9%), which in the majority of cases have led to a diagnosis. In addition, neck stiffness, fever and coma completed the diagnosis;
- Neuromeningeal cryptococcosis was also found in people living with HIV with a CD4 count > 200 cells/mm^3^ (8.7% of cases). In addition, a predominantly neutrophil-dominant leukocyte pattern in the CSF was found in most patients (73.9%), suggesting possible bacterial co-infection.
